# Networks Models of Actin Dynamics during Spermatozoa Postejaculatory Life: A Comparison among Human-Made and Text Mining-Based Models

**DOI:** 10.1155/2016/9795409

**Published:** 2016-08-24

**Authors:** Nicola Bernabò, Alessandra Ordinelli, Marina Ramal Sanchez, Mauro Mattioli, Barbara Barboni

**Affiliations:** ^1^Faculty of Veterinary Medicine, University of Teramo, Via Renato Balzarini 1, 64100 Teramo, Italy; ^2^Istituto Zooprofilattico Sperimentale dell'Abruzzo e del Molise “G. Caporale”, Campo Boario, 64100 Teramo, Italy

## Abstract

Here we realized a networks-based model representing the process of actin remodelling that occurs during the acquisition of fertilizing ability of human spermatozoa (HumanMade_ActinSpermNetwork, HM_ASN). Then, we compared it with the networks provided by two different text mining tools: Agilent Literature Search (ALS) and PESCADOR. As a reference, we used the data from the online repository Kyoto Encyclopaedia of Genes and Genomes (KEGG), referred to the actin dynamics in a more general biological context. We found that HM_ALS and the networks from KEGG data shared the same scale-free topology following the Barabasi-Albert model, thus suggesting that the information is spread within the network quickly and efficiently. On the contrary, the networks obtained by ALS and PESCADOR have a scale-free hierarchical architecture, which implies a different pattern of information transmission. Also, the hubs identified within the networks are different: HM_ALS and KEGG networks contain as hubs several molecules known to be involved in actin signalling; ALS was unable to find other hubs than “actin,” whereas PESCADOR gave some nonspecific result. This seems to suggest that the human-made information retrieval in the case of a specific event, such as actin dynamics in human spermatozoa, could be a reliable strategy.

## 1. Introduction

Postgenomic era offers to researchers amazing opportunities in approaching a myriad of biological problems. One of the most interesting issues is the use of computational models for representing and analysing complex biological systems. They make researchers able to face important problems, such as those arising from the availability of a huge amount of data to be analysed (the so-called big data challenge) and from the creation of new information from the already available published data. This last issue, on one hand, is very timely and offers fascinating horizons, whereas, on the other one hand, it requires further studies to verify the reproducibility and the reliability of the obtained data. In this context, here we focused our attention on a biological event, which has a great importance in spermatology and in applied andrology: the dynamics of actin during the postejaculatory life of male gametes. Indeed, immediately after ejaculation, mammalian spermatozoa are virtually unable to fertilize the homologous oocyte. They become fully fertile only after they reside for hours to days within the female genital tract, where they complete a complex process of functional maturation known as capacitation. During capacitation spermatozoa biochemical machinery changes its function as a result of the dialogue between male gametes and female environment (tubal epithelium, tubal fluid, and female endocrine axis). The ionic intracellular concentration of ions changes, the protein phosphorylation is modified, sperm motility becomes hyperactivated, and plasma membrane (PM) and outer acrosome membrane (OAM) became gradually more fluid and tend to fuse each other. In this context, to date, it is believed that immediately after ejaculation the actin present in sperm head is mainly in globular unpolymerized form (G-actin). As the capacitation progresses, the actin undergoes polymerization, forming a network of F-actin that interposes between outer acrosome membrane (OAM) and plasma membrane (PM), thus avoiding their premature fusion. When the physiological stimulus of acrosome reaction, the zona pellucida proteins, is met, this diaphragm is destroyed and the two membranes can fuse. Recently it has been suggested that the role of actin dynamic in this context could go beyond the merely mechanical function, but that this protein could be involved in the pathway as an active signal transducer [1].

From this point of view, it will be very interesting to have available a computational model of actin dynamics during the postejaculatory life of spermatozoa. At the present, a specific model devoted to the representation of actin dynamics during capacitation life is not already available; thus we carried out a study comparing a new model based on the manual compilation of a database, analogously to other database that we have already realized [2, 3] with ones obtained by a text mining-based approach. We paid our attention to text mining because it represents a new, important, and fascinating resource for information retrieval [4] and for constructing interaction network from biomedical texts [5]. Recently, this approach has been adopted to explore the biology of different phenomena, such as the prostate cancer protein interaction network, by using a reinforcement learning-based algorithm [5], or in studying other types of tumours [6–9] and physiological [10–12] and pathological events [13–15]. Here, in detail, we realize a model, starting from the analysis of published literature on this topic and we compared it with models realized by two different text mining tools, able to produce networks: Agilent Literature Search and PESCADOR. As a reference, we used the data from the online repository KEGG (Kyoto Encyclopaedia of Genes and Genomes), which are referred to the actin polymerization and depolymerisation in a wide variety of cells and not specifically to the spermatozoa.

## 2. Materials and Methods

### 2.1. Data Collection, Network Creation, and Analysis

#### 2.1.1. Human-Made Spermatozoa Actin Network (HM_SAN)

In this work, we used different networks. The first was realized by considering the scientific literature published in peer-reviewed international papers indexed in PubMed archive (http://www.ncbi.nlm.nih.gov/pubmed/) in the last 15 years [2, 3]. As reference, we used the data referred to human species. Following an already validated protocol [16], two researchers expert on spermatozoa biology carried out an independent literature analysis on papers using the following key words: “Actin polymerization”, “Actin depolymerisation”, “Actin dynamics”, and “Actin remodelling”. Then, the two databases have been compared, and a third researcher verified the correctness of the record inserted and resolved eventual conflicts. The freely available and diffusible molecules such as H_2_O, CO_2_, P_*i*_, H^+^, and O_2_ were omitted, when not necessary, and in some cases the record did not represent a single molecule but a complex event, such as “protein tyrosine phosphorylation” because all the single molecular determinants of the phenomenon are still unknown [10, 17].

This database (interaction database), was realized in Microsoft Excel 2013 and contained the following fields:

- *Source molecule*: here are reported the molecules source of the interaction.
- *Interaction*: here is described what kind of interaction the molecules carry out.
- *Target molecule:* here are reported the molecules that are target of the interaction.
- *Alias*: eventual aliases are described.
- *Role*: the physiological and/or pathological role of the molecule in epididymis is reported.
- *Reference*: it represents the paper reporting the above mentioned data.
- *Notes*: any further information that could be useful in the study is mentioned here.

#### 2.1.2. Agilent Literature Search-Spermatozoa Actin Network (ALS_SAN)

This network was realized by using Agilent Literature Search Software, a metasearch tool for automatically querying multiple text-based search engines that can be used in conjunction with Cytoscape, thus generating a network view of protein associations. In particular, we used the Cytoscape 3.3.0. App Agilent Literature Search 3.1.1 beta (LitSearch version 2.69), using as data source the papers contained in PubMed database. As key words, we used the same key words used to build HM_ASN, using as context “spermatozoa”. Max Engine Matches was set at 1.000 (which always was higher than the number of articles found; thus in all the cases all the available information was processed); the “Use Aliases,” the “Use Context,” and the “Concept Lexicon Restrict Search” options were set. As Concept Lexicon “Homo sapiens” we used. The data have been accessed until April 15, 2016. We created ALS_SAN by merging all the obtained networks and removing self-loops and the duplicated edges [10].

#### 2.1.3. PESCADOR-Spermatozoa Actin Network (P_SAN)

This network was created by using PESCADOR (Platform for Exploration of Significant Concepts AssociateD to co-Occurrence Relationships), which is a platform independent web resource (http://cbdm.mdc-berlin.de/tools/pescador/) [18]. It analyses a query composed of a list of PMIDs to be scanned for gene/protein cooccurrences and, optionally, of a list of words (ideally, biological concepts related to protein interactions, such as “aggregation” or “phosphorylation”) to be found in the cooccurrence analysis, as text mining engine to extract sentences with cooccurring bioentities from the text of the PubMed abstracts requested that it uses LAITOR (Barbosa altro). P_SAN was created by using the list of PMIDs of the papers we have manually selected for the realization of HM_SAN.

### 2.2. KEGG_AN

This network, used as reference, has been created by importing the data from KEGG (Kyoto Encyclopaedia of Genes and Genomes), a database resource for understanding high-level functions and utilities of the biological system, and from molecular-level information, especially large-scale molecular datasets generated by genome sequencing and other high-throughput experimental technologies (http://www.genome.jp/kegg/). We analysed the data from the pathway: map04810—regulation of actin cytoskeleton. This network is not specifically designed to represent the actin dynamics occurring during sperm capacitation, but it is generically referred to the actin cytoskeleton rearrangement. It was used to compare the other networks with a network representing a strongly related biological event and certified by a rigorous quality control [19, 20].

### 2.3. Networks Visualization and Analysis

All these networks have been realized, visualized, and analysed using Cytoscape 3.1.2 [21]. The analysis was carried out considering the networks as undirected and assessing the topological parameters listed and described in Table 1.

To represent the nodes as Venn's diagram, we used Venny, a specific tool, available at http://bioinfogp.cnb.csic.es/tools/venny/.

### 2.4. Network Randomization

To compare our networks with a computer-generated network following the Barabasi-Albert model, we used the Cytoscape plug-in Network Randomizer 1.1 (http://apps.cytoscape.org/apps/networkrandomizer). We used the Barabasi-Albert model and we set the parameters *N* = 128 and *m* = 2. We obtained the Barabasi_Albet random network (BA_RN) constituted by 2 connected components of, respectively, 125 (main component, BA_RN, and MC_BA_RN) and 3 nodes.

## 3. Results

We obtained five different networks: HM_SAN, P_SAN, ALS_SAN, KEGG_AN, and BA_RN. The results of their topological analyses are shown in Table 2, where the values of main topological parameters are listed. In the case of the network obtained by using PESCADOR, we found that it contained several nonspecific nodes (such as “acrosome”, “spermatozoa”, “membrane”, and “in vitro”). After their removal, we obtained P_ASN and a second network, its main connected component, MC_P_ASN. Also in the case of ASL_SAN, KEGG_AN, and BA_RN we extracted the main connected components (MC_ALS_SAN, MC_KEGG_SAN, and MC_BA_RN). In Table 3 are reported the results of the fitting of node degree versus the number of nodes. In Table 4 are shown the results of the correlation analysis between the node degree and the clustering coefficient of all the networks. In Table 5 are listed the hubs of the networks. In Supplementary Material (available online at http://dx.doi.org/10.1155/2016/9795409) are listed the articles we used to build our database and those used by ALS, highlighting the common ones.

## 4. Discussion

**Figure 1:**
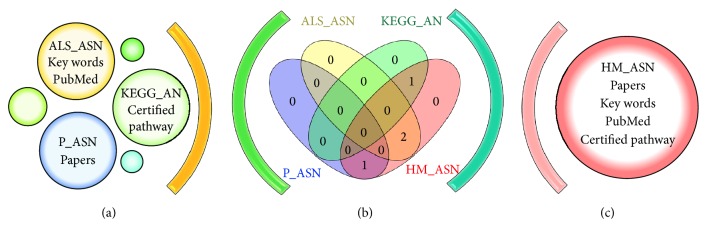
(a) In yellow are represented the search parameters that our database, HM_ASN, has in common with the ALS_ASN software, in blue with P_ASN software (two text mining tools), and in green with the KEGG database. (b) Venn diagram representation, which allowed the identification of the intersections between different databases other than HM_ASN. (c) In red are represented the elements used to create the database HM_ASN.

**Figure 2:**
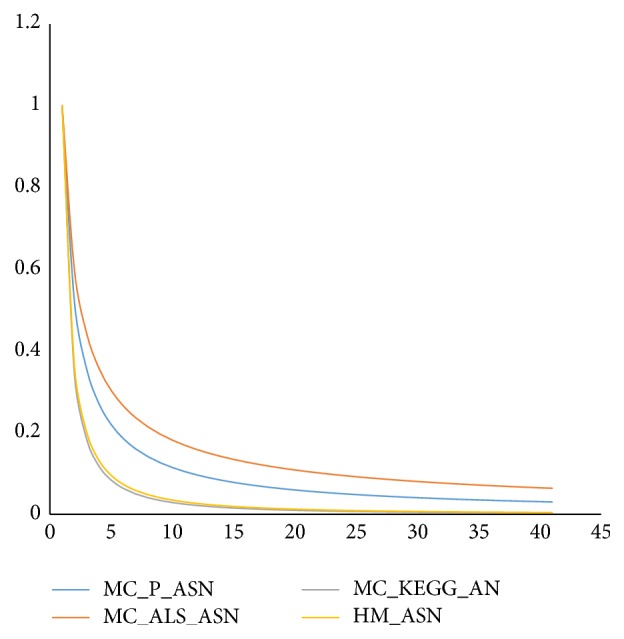
Curves that represent the node degree distribution in HM_SAN, MC_P_ASN, MC_ALS_ASN, and MC_KEGG_AN.

**Figure 3:**
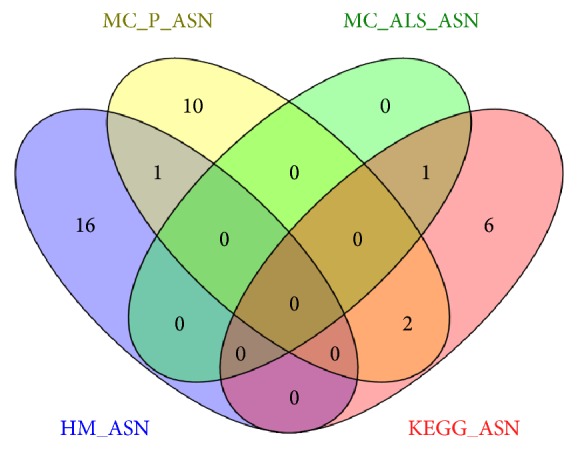
Venn's diagram showing the common hubs in HM_SAN, MC_P_ASN, MC_ALS_ASN, and MC_KEGG_AN.

Here, we realized a network representing actin dynamics during sperm capacitation (HM_ASN); then we compared it with two networks generated by two text mining software, able to directly provide networks models (P_ASN and ALS_SAN). As reference we used a peer-reviewed and quality controlled network (KEGG_AN) related to the same biological event, but it referred to a more general context and a Barabasi-Albert scale-free network generated by the computer (BA_RN). See Figure 1. From our analysis, it is clear that HM_SAN has a scale-free topology, in keeping with the Barabasi-Albert model. Indeed, it is very close to BA_RN and it has the node degree (i.e., the number of nodes per link) probability distribution following an exponential law with a negative exponent and uncorrelated with the clustering coefficient (which represents the network tendency to develop clusters). In addition, the network has a small world topology, as evident from the values of shortest paths (100%), characteristic path length, and averaged number of neighbors (4.064 and 2.921, resp.). These measures suggest that the information is spread within the network in a very fast and efficient way and that the network is able to quickly adapt to the external perturbations. In particular, the low value of clustering coefficient indicates that loop or clusters, that could interfere and slow the propagation of messages, are virtually absent in HM_ASN. KEGG_SN has virtually the same topology of HM_ASN, thus suggesting that the network we created could be representative of a similar biological event, and that this pattern could be typical of signalling pathways. This finding is in accordance with those we have found when analysing several other networks referred either to sperm signalling or to other biologically relevant events. Indeed, recently, we compared the networks representing the biochemical machinery involved in spermatozoa in sea urchin,* Caenorhabditis elegans*, and human male gametes, with networks representing ten pathways of relevant physiopathological importance and with a computer-generated network [22]. As a result, we have found that all the networks studied are characterized by robustness against random failure, controllability, and efficiency in signal transmission. In all the cases, the clustering coefficient had values near zero [22]. Interestingly, the two networks generated by the text mining software have a different topology. Both of them are characterized by a lower absolute value of exponent of node degree distribution (see Figure 2) and by a higher value of clustering coefficient, whose distribution correlated with the node degree, as shown in Table 4. Then they could be considered hierarchical networks. This finding highlights that ALS and PESCADOR seem to give results not completely comparable with those from manual compilation of databases. This idea is also highly strengthened by the analysis of networks hubs. Indeed, the scale-free topology of all the networks allows one to identify the nodes exerting the higher level of control within the network, the hubs, calculated as the nodes with a node degree with a degree at least one standard deviation above the network mean [23]. As it is reported in Table 5 we found great differences either in number or in identity among the hubs from the different networks. Interestingly the only hub shared by all the networks is “actin” (see Figure 3).

The hubs of HM_ASN are F-actin and complex events related to the signal transduction pathway involved in actin remodelling occurring during the process of spermatozoa acquisition of fertilizing ability such as “Actin polymerization” and “Actin depolymerization”, or proteins “Tyrosine phosphorylation”. In addition we have identified as hubs several molecules involved in input of control messages (EGFR, H_2_O_2_, and HCO_3_^−^), second messengers ([Ca^2+^]_*i*_, cAMP, ROS, and PIP2 cleavage), and effector molecules (PKC, PLD, Rho GTPase, Arp2/3 complex, and ADF/cofilin). This finding is in agreement with the currently proposed model of actin signalling transduction pathway active in human and mammalian spermatozoa, based on experimental data. Indeed, the actin dynamics occurring during capacitation and acrosome reaction are under the control of several activating factors. The most important extracellular activating messenger is thought to be the bicarbonate [24–27], which is able to enter the cells and to stimulate the production of cAMP, via the activation of a specific soluble adenylyl cyclase (sAC). The rise in intracellular level of cAMP leads to the increase in membrane scrambling and directly or indirectly causes the increase in cytosolic concentration of the other second messengers: Ca^2+^ [28, 29], cAMP [30], ROS [17, 31, 32], and DAG and IP3 (resulting from PIP2) [33, 34]. This promotes the activation of a myriad of cellular effectors that directly and indirectly control the actin polymerization status [35, 36]. In particular, it has been demonstrated that PKA, PKC, and PLD1 play a key role in modulating the actin polymerization/depolymerisation status [35, 37, 38]. KEGG_ASN contains several proteins involved in cell signalling, such as RAC1, ROCK1, PAK4, RHOA, CDC42, ARHGEF7, MYL12B, and RRAS2, and virtually all those involved in Rho signalling and, of course, it is known to participate in actin cytoskeleton remodelling (see for reference [39]).

More interestingly, ALS_SAN contains only one hub: actin. This could be explained with the search logic of ALS that, likely, is able to consider only the molecules directly interacting with actin, thus excluding from the results indirect relationships, which were instead took into account by human database compilers. This reason will explain also the hierarchical structure network. We examined also the papers identified by human manual compilers of database and those identified by ALS. We have found 26 papers related to the used key words and published in last 15 years suitable to be used to gather information about actin dynamics. ALS identified 31 papers, 4 of which have been published before this range of time; see Supplementary Material. Twelve papers have been identified by both the systems; the others differ. This difference could be, in our opinion, explained with two hypothesis:

- Human compilers discarded similar papers (mainly reviews) from the same group, using only the most recent ones.
- Human compilers included also papers, which did not have “actin” in key words, expanding the selection criteria.

PESCADOR gives a high number of hubs, actually corresponding to proteins involved in actin signalling. Curiously, it considers also MSP, the Major Sperm Protein, which is involved in spermatozoa cytoskeleton signalling, but in Nematoda that lack actin [40].

## 5. Conclusions

In conclusion, we could affirm that

- HM_ASN and KEGG_AN are very similar, in terms of topology; this could suggest that the human information retrieval in the case of a specific event, such as actin dynamics during mammalian spermatozoa, could be a reliable strategy;
- PESCADOR seems to give nonspecific results that need to be manually removed from the model; thus the reliability of their results needs to be improved;
- ALS tends to be less “elastic” than human retrieval; indeed it collects only the data strictly related to the actin, leaving out the molecules indirectly interacting with actin.

It is possible to hypothesize that when searching for a very specific query the human bases research could offer more reliable data, in comparison with text mining tools. Likely, these systems could be needed when the number of papers to be checked is larger.

## Supplementary Material

Here are listed the publications that result from our PubMed search and those find by ALS.

## Figures and Tables

**Table 1:** Main topological parameters assessed in this study.

Parameter	Definition
Connected components	It is the number of networks in which any two vertices are connected to each other by links and which is connected to no additional vertices in the network.
Number of nodes	It is the total number of molecules involved.
Number of edges	It is the total number of interactions found.
Clustering coefficient	It is calculated as *CI* = 2*nI*/*kI*(*kI* − 1), where *nI* is the number of links connecting the *kI* neighbors of node *I* to each other. It is a measure of how the nodes tend to form clusters.
Network diameter	It is the longest of all the calculated shortest paths in a network.
Shortest paths	The length of the shortest path between two nodes *n* and *m* is *L*(*n*, *m*). The shortest path length distribution gives the number of node pairs (*n*, *m*) with *L*(*n*, *m*) = *k* for *k* = 1,2,….
Characteristic path length	It is the expected distance between two connected nodes.
Averaged number of neighbors	It is the mean number of connections of each node.
Node degree	It is the number of interactions of each node.
Node degree distribution	It represents the probability that a selected node has *k* links.
*γ*	Exponent of node degree equation.
*R* ^2^	Coefficient of determination of node degree versus number of nodes, on logarithmized data.

**Table 2:** Results of topological analyses of networks.

Parameter	P_ASN	MC_P_ASN	ALS_ASN	MC_ALS_ASN	KEGG_AN	MC_KEGG_AN	HM_ASN	BA_RN	MC_BA_RN
Connected components	10	1	7	1	23	1	1	2	1
Number of nodes	136	109	86	66	84	60	128	128	125
Number of edges	283	234	161	141	75	73	187	196	194
Clustering coefficient	0.577	0.552	0.637	0.656	0.017	0.024	0.073	0.024	0.025
Network diameter	9	9	5	5	9	9	10	10	10
Shortest paths	11624 (63%)	8230 (69%)	4344 (59%)	4290 (100%)	3546 (50%)	3540 (100%)	16256 (100%)	13812 (95%)	13806 (100%)
Characteristic path length	3.799	3.876	2.332	2.346	4.294	4.299	4.064	4.440	4.441
Avg. number of neighbors	4.162	4.294	3.744	4.273	1.786	2.433	2.921	2.975	3.017

**Table 3:** Results of node degree analysis of networks.

	P_ASN	MC_P_ASN	ALS_ASN	MC_ALS_ASN	KEGG_AN	MC_KEGG_AN	HM_ASN	BA_RN	MC_BA_RN
*γ*	−1.348	−0.941	−0.881	−0.741	−1.596	−1.540	−1.459	−1.317	−1.299
*r*	0.741	0.825	0.862	0.790	0.530	0.494	0.979	0.895	0.860
*R* ^2^	0.736	0.546	0.671	0.617	0.799	0.778	0.860	0.805	0.780

**Table 4:** Results of fitting on node degree versus clustering coefficient.

	P_ASN	MC_P_ASN	ALS_ASN	MC_ALS_ASN	KEGG_AN	MC_KEGG_AN	HM_ASN	BA_RN	MC_BA_RN
*γ*	−0.763	−0.479	−0.915	−0.921	−0.178	−0.198	−0.490	−0.640	−0.663
*r*	0.737	0.662	0.704	0.708	0.121	0.128	0.647	0.414	0.438
*R* ^2^	0.477	0.342	0.810	0.815	0.030	0.038	0.505	0.482	0.391

**Table 5:** Hubs of the networks.

HM_ASN	MC_P_ASN	MC_ALS_ASN	KEGG_ASN
PKA	RHOA	ACTIN	RAC1
Actin polymerization	MSP		ROCK1
Tyrosine phosphorylation	EGFR		PAK4
[Ca^2+^]_*i*_	LIMK		RHOA
cAMP	CDC42		CDC42
ROS	GNA13		ACTIN
Actin depolymerisation	ROCK2		ARHGEF7
F-actin	LIMK2		MYL12B
PLD	ACE		RRAS2
Rho GTPase	AKAP4		
H_2_O_2_	AKAP3		
PIP2 cleavage	PRKAR2		
Arp2/3 complex	ROPN1		
ADF/cofilin			
EGFR			
HCO_3_^−^			
PKC			
